# A Systematic Review Investigating the Presence of Inflammatory Synovitis in Hip and Knee Joint Replacement Surgery

**DOI:** 10.1155/2015/729410

**Published:** 2015-10-18

**Authors:** Sherif Hosny, Francesco Strambi, Nidhi Sofat, Richard Field

**Affiliations:** ^1^South West Thames Training Rotation, London, UK; ^2^South West London Elective Orthopaedic Centre, Dorking Road, Epsom, Surrey KT18 7EG, UK; ^3^St George's, University of London, Cranmer Terrace, London SW17 0RE, UK; ^4^Epsom & St Helier NHS Trust, UK

## Abstract

Synovial tissue can display an inflammatory response in the presence of OA. There is increasing interest to better understand the role of inflammation in OA, particularly with regard to those who require joint replacement. A systematic review of inflammatory synovitis in OA of literature databases was undertaken from their inception until October 14, 2014. Independent critical appraisal of each study was undertaken using the CASP appraisal tool. From a total of sixty-six identified citations, twenty-three studies were deemed eligible for review. The studies presented moderate to strong methodological quality. Strong correlation was identified between histological and imaging synovitis severity. Correlation was weaker between clinical symptoms and imaging and/or histological synovitis severity. There was little consensus, with regard to expressed cytokines and chemokines at the different stages of OA disease progression. Few studies investigated the influence of inflammatory synovitis on the outcome of major joint replacement. Research into inflammatory synovitis in OA is an emerging field. Longitudinal studies applying proven imaging modalities, histological analysis, and longer follow-up are required in order to further define our understanding of the role of synovitis in the pathogenesis of OA and its effects on outcomes following major joint replacement.

## 1. Introduction

People with osteoarthritis (OA) typically experience joint pain, stiffness, and swelling. The condition causes progressive physical disability and pain. The aetiology of OA is multifactorial [1], with both systemic and local biomechanical factors identified [2]. The prevalence of OA in the US is estimated at nearly 27 million people and accounts for 25% of visits to primary care physicians [3]. In the hip and knee, OA often causes progressive joint damage with growing numbers requiring joint replacement surgery [4].

In contrast to rheumatoid arthritis (RA), OA has conventionally been considered a noninflammatory condition. This may be simplistic. The term arthritis was coined to describe joint inflammation. Trainees have long been taught that synovial inflammation and joint effusion are common in the early stages of degenerative joint disease and synovial proliferation is a common finding in patients undergoing arthroscopy and arthroplasty [5]. Magnetic Resonance Imaging (MRI) has identified synovitis in early OA, even in the absence of the relevant clinical findings [6]. In the wake of recent successes in the treatment of RA, attention is now turning to the possibility of medical treatments to suppress or slow the inflammatory elements of OA.

At a cellular level, the role of immune cells and cytokines is well established. Infiltration of macrophages and perivascular T and B lymphocytes is described in early and advanced disease [7]. The main cytokines identified in OA pathogenesis are Interleukin-1*β* (IL-1*β*) and Tumour Necrosis Factor *α* (TNF*α*) [8]. It is known that these cytokines can mediate the production of other cytokines, matrix metalloproteinases (MMP), and proteases with effects on T lymphocytes, chondrocytes, and synovial cells.

The purpose of this systematic review is to identify studies demonstrating the inflammatory changes that are frequently observed in patients undergoing joint arthroplasty and the influence of the synovial response on disease progression and treatment outcome.

## 2. Material and Methods

### 2.1. Search Strategy

One reviewer (Sherif Hosny) performed a PRISMA compliant search of the electronic databases EMBASE and Medline and Cochrane via the Ovid platform from inception until October 14, 2014. In addition, grey literature and trial registry searches were conducted using the WHO International Clinical Trials Registry Platform, Current Controlled Trials, and the United States National Institute of Health Trials Registry. The NIHR Clinical Research Portfolio Database was searched as was the ISI Web of Knowledge and OpenGrey System for Information on Grey Literature in Europe. The search terms adopted included “exp Synovitis” OR “spondyloarthropathy/” OR “exp HLA-B27 Antigen/” OR “synovitis.mp.” OR “seronegative.mp.” AND exp arthroplasty/OR hip replacement^*∗*^.mp OR knee replacement^*∗*^.mp OR arthroplasty.mp. OR joint replacement^*∗*^.mp. An English language restriction was applied. Additionally, the reference lists of all identified articles were screened for additional papers.

### 2.2. Inclusion/Exclusion Criteria

Study identification was initially performed by one reviewer (Sherif Hosny) and then verified by another (Francesco Strambi) after consulting the titles and abstracts. The search strategy was run and identified articles were exported as titles and abstracts. Papers were then retrieved in full and a further round of relevancy screening was undertaken by the two reviewers (Sherif Hosny and Francesco Strambi). Studies presenting nonoriginal data, such as reviews, editorials, opinion papers, and letters to the editor, were excluded. Conference presentation abstracts with no retrievable data were excluded. Studies with nonhuman subjects were excluded. Studies in paediatric subjects were excluded. For all papers initially considered eligible, full texts were ordered, and those satisfying the eligibility criteria were included in the final review.

### 2.3. Methodological Appraisal

All studies identified in the search strategy and included in this review were assessed using the (Critical Appraisal Skills Programme) CASP cohort study appraisal tool. Eleven critical appraisal questions were asked of each paper. These are itemized in Table 1 and assess the internal and external validity of each included study. Each study was evaluated against this checklist by one reviewer (Sherif Hosny) and verified by a second (Francesco Strambi). Any disagreements were resolved through consensus.

## 3. Results

### 3.1. Search Results

**Figure 1:**
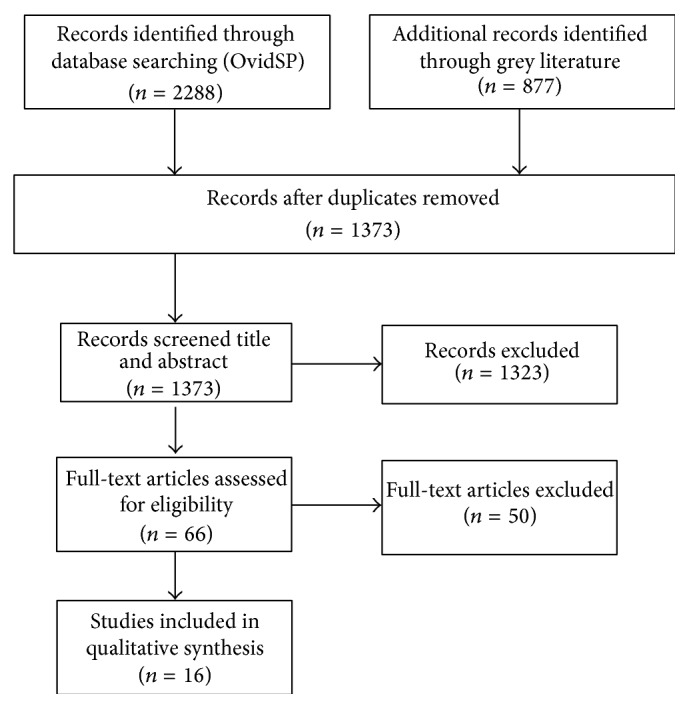
PRISMA flowchart.

Initial search yielded 3165 papers from the search strategies. These were all exported as titles and abstracts. After screening the titles and abstracts for relevancy, 1323 citations were excluded. A further round of relevancy screening was undertaken and 66 papers were identified for retrieval. A final tally of 16 papers were reviewed (Figure 1).

### 3.2. Methodological Quality

The results of the CASP critical appraisal are shown in Table 1 and presented moderate to strong methodological quality. The included papers always addressed a focused issue and recruited in a replicable and acceptable way. All papers described their cohort's characteristics and trial eligibility criteria. Exposure and outcome bias was reasonably well controlled. All studies provide sufficient reporting to permit generalizability of their results to a clinical population. However, recurrent limitations in the evidence base included poor identification of important confounders (*n* = 5) and not accounting for confounding variables in the design of the study (*n* = 7). Some studies reported results that contradicted other available evidences or there was no other published evidence to compare to (*n* = 3).

### 3.3. Study Characteristics

A summary of the included study characteristics is presented in Table 2. Eight studies were concerned with imaging of the synovitis using either MRI or ultrasound and the correlation of imaging with histopathological grading. Seven studies were concerned with detailed histological analysis of the synovitis. One study evaluated outcomes associated with synovitis.

### 3.4. Clinical Findings

#### 3.4.1. Correlation of Synovitis Severity in Imaging with Histological Severity

Three authors found significant correlations between synovitis, as seen on MRI, and histological severity grading [9–11]. In the first of these, Østergaard et al. [9] used gadolinium enhanced MR scanning and showed that while dynamic MRI could distinguish knees with and without synovial inflammation, it could not differentiate between moderate and severe inflammation. De Lange-Brokaar et al. [10] used 3 T gadolinium enhanced MR scanning in 41 patients, undergoing Total Knee Replacement (TKR) or arthroscopy, and found significant correlation between total synovitis grade using the Guermazi grading system [12] and the total histology grade using a modified Krenn system [13]. Similarly Liu et al. [11] found significant correlation between gadolinium enhanced MRI synovitis score and total synovitis score on histological analysis using a similar grading system. A positive association was also found with macroscopic features such as neovascularisation, hyperplasia, and villi formation.

Power Doppler US (PDUS) was the focus of three studies in the literature [14–16]. Walther et al. [14, 15] used PDUS to image osteoarthritic hips and knees prior to arthroplasty. In both joints, PDUS proved to be reliable in qualitative grading of the vascularity of the synovial tissue as graded after staining with haematoxylin and eosin or factor VII (immunohistochemistry). Interestingly, there was a correlation between the thickness of the synovial membrane and the PDUS signal but no correlation between synovial proliferation and effusion.

In a study by Takase et al. [16], grey scale ultrasound scan (GSUS), PDUS, and contrast enhanced MRI were evaluated against histopathology findings in OA and RA patients undergoing TKR. In this study,* cluster of differentiation 68* (CD68) was used as a marker for inflammatory cell infiltrate,* antigen Ki-67* for synovial lining cell thickness, and* cluster of differentiation 31* (CD31) for vascularity. All three imaging modalities showed a positive correlation with the histopathological total synovitis score with PDUS showing the highest correlation. In contrast, only PDUS and MRI showed high correlation with the immunohistochemical parameters investigated.

Conversely, Waldstein et al. [17], using 1.5 T or 3 T MRI, without contrast, found no correlation between MRI synovitis grades and histopathological grades using validated scoring systems. The WORMS system [12] was used for MRI synovitis grading and the Krenn system [13] for histopathological grading. While the correlation between imaging and histological synovitis was not the core focus of this study, the authors did find a relationship between intra-articular inflammation and cartilage properties (see Section 3.4.4).

The use of MRI, MRS, and PDUS has been described in recent studies to measure synovitis, which could account for some differences between observed studies. It is therefore important that future studies utilise standardised tools for measuring synovitis radiographically. It has been suggested that MRI techniques, especially with contrast, could be optimal because standardised views can be reproduced and synovitis can be most easily differentiated from other vascular structures.

#### 3.4.2. Correlation of Histological or Imaging Severity with Clinical Findings

Only a few workers have tried to correlate synovitis with pain. De Lange-Brokaar et al. [18] found a significant correlation between MRI synovitis score and pain level as recorded, on a visual analogue scale (VAS), in knee osteoarthritis patients undergoing TKR or arthroscopy. The same authors in an earlier paper [19] found no association between MRI synovitis score and pain. However, the earlier study had fewer participants and included only patients due for arthroscopy and not TKR. In the later work, the authors reported a positive association between synovitis and pain as measured by VAS or Knee Injury and Osteoarthritis Score (KOOS) or Intermittent and Constant Osteoarthritis Pain (ICOAP) scores. However, these findings depended on the location of the synovitis within the knee. It was observed that different synovitis locations correlated with different pain scoring systems. In contrast, in a study of 34 Japanese patients with OA requiring TKR, Liu et al. [11] found that neither the synovitis scores evaluated by histological analysis nor those by a gadolinium enhanced MRI scan were correlated with pain as assessed on the VAS or the Western Ontario and McMaster Universities Osteoarthritis Index (WOMAC) score [20]. They did however correlate with the Japanese Knee Osteoarthritis Measure (JKOM) [21] score.

#### 3.4.3. Early versus Late Osteoarthritis

Three studies have investigated differences in synovial tissue inflammation in early and late OA. Benito et al. [22] compared patients with advanced OA, awaiting TKR, against patients with early OA. Synovial tissue from early OA patients exhibited greater lining layer thickness as well as greater intensity of* CD4+* T cell and* CD68+* macrophage infiltration. Blood vessels were more numerous as were vascular endothelial growth factor (VEGF), a marker of vascular proliferation, and intercellular adhesion molecule 1 (ICAM-I), expressed on vascular endothelial cells. Greater numbers of cells producing TNF*α* and IL-1*β* were seen in early OA. However, fibroblast like synoviocytes were functionally similar in both groups. In another study by Liang et al. [23], end stage OA patients, undergoing TKR, were compared with Anterior Cruciate Ligament (ACL) reconstruction patients. Matrix metalloproteinase-1 (MMP-1), cyclooxygenase COX-2, and IL-1*β* expression were significantly greater in those with progressive OA as compared to those with end stage disease. Transforming growth factor beta (TGF*β*) expression was found to be greater in end stage OA.

Conversely Scanzello et al. [24] found differences only in IL-15 levels between early OA and late OA. Patients with end stage OA undergoing TKR had significantly lower levels than patients undergoing arthroscopy for meniscal tears. In another study, Richardson et al. [25] compared levels of Interleukin-8 (IL-8), IL-1*β*, Interleukin-6 (IL-6), Interleukin-10 (IL-10), TNF, and Interleukin-12 (IL-12) in synovial fluid samples from OA, RA, and normal patients. Only IL-6 was significantly higher in the RA and OA samples in comparison to the normal group.

De Lange-Brokaar et al. [10] found that more patients in their arthroplasty group showed inflammatory infiltrates compared to their arthroscopy group. Furthermore, the grades for both the lining layer and the inflammatory infiltrate were significantly higher in the arthroplasty group. For the stroma, no significant differences were identified between the groups. These findings supported an earlier study by Smith et al. [26] which showed synovial membrane inflammation to be more severe, exhibiting a thicker lining layer, increased vascularity, and more abundant inflammatory cell infiltrate with advanced OA. Increased levels of IL-1*α*, IL-1*β*, and TNF*α* were also seen with increased cartilage damage.

Oehler et al. [27] categorised the synoviopathy present in OA into four distinct categories depending on the histomorphological features observed. In early OA, a mostly hyperplastic subtype was observed. This was distinguished from other variants by its villous hyperplasia and relative absence of inflammatory infiltrates, capsular fibrosis, and cartilage and bone detritus. An inflammatory subtype, characterised by synovial hyperplasia and moderate lymphocytic infiltrates, was seen in early and late OA. This subtype was also noted for its lack of capsular fibrosis and cartilage debris. Late stage OA was also seen in what were deemed detritus rich and capsular fibrosis subtypes. These were characterised by cartilage and bone debris and capsular fibrosis, respectively.

#### 3.4.4. Cartilage Effects

In a biomechanical study of the effects of inflammation on cartilage integrity, De Lange-Brokaar et al. [18] found that lateral compartment cartilage was mechanically inferior in knees undergoing TKR for OA in the presence of a white cell count >150 WBC/mL. These cartilage samples had significantly reduced mean aggregate and dynamic modulus when compared to samples taken from knees with <150 WBC/mL. However, in contrast to the majority of other studies, Waldstein did not identify any link between MRI synovitis grade and aggregate or dynamic modulus or link between MRI synovitis grade and histopathologic grade.

#### 3.4.5. Gene Expression

Lambert et al. [28] identified 896 genes that were differently expressed between two areas of synovial membrane from the same patient. In this study, twelve patients undergoing knee replacement were investigated. Synovial tissue was macroscopically categorised according to the Ayral criteria [29] as either normal/reactive or inflamed at the time of operation by the surgeon. Genes for inflammatory cytokines, chemokines, anabolism compounds, catabolism compounds, and angiogenesis compounds were upregulated in areas of inflamed synovium compared to uninflamed control areas. These key pathways were related to inflammation, cartilage metabolism, wingless-related integration site (Wnt) signalling, and angiogenesis. Specific compounds are listed in Table 3.

#### 3.4.6. Outcomes

Tanavalee et al. [30] have investigated the effect of synovectomy in TKR. In two similar groups for which there was no observed difference in inflammatory synovitis at operation, it was shown that synovectomy made no difference neither to clinical outcome score, as measured by the American Knee Society Score [31], or to serial postoperative inflammatory markers as measured by ESR, CRP, IL-6, or mean skin temperature. As such, the authors concluded that synovectomy at the time of TKR does not improve clinical outcome or shorten the duration of the inflammatory response after surgery.

## 4. Discussion

We have identified an expanding body of published literature on synovitis in OA. This reflects a growing acceptance that OA can no longer be considered a purely noninflammatory condition and that synovitis is prevalent in OA patients. The purpose of this review was to identify the pathophysiology of the synovitis found in OA and to investigate the effect of this synovial response on risk of progression and treatment outcomes. In order to focus this study further, we assessed only papers that included an intervention in the studied population such as TKR or THR. Previous reviews have focused on synovitis pathology in general or in the context of OA but have not correlated imaging with histology or assessed the correlation between presence on imaging and/or histology with clinical symptoms.

We have observed a strong correlation between imaging of the synovitis on MRI and histological findings. However, there were two exceptions to this trend. In the Østergaard et al. [9] study, MRI could not differentiate between moderate and severe inflammation. This may be explained by the fact that most patients in the study, in contrast to other studies, had advanced disease with minimal overlap between histologic groups. Thus, the differentiation between moderate and severe inflammation was less distinct. In De Lange-Brokaar et al.'s [19] study, MRI did not correlate with histological findings. They used the validated Krenn et al. scoring system used by other authors but did not use contrast enhanced MRI as most other authors did.

The authors, Walther et al. [14, 15] and Waldstein et al. [17], found that PDUS correlates well with histological grade and has been shown to provide better correlation than MRI with total synovitis score. As a consequence, these authors have advocated the use of PDUS to detect joint synovitis.

There is less evidence for correlation of imaging or histological severity with clinical findings. In Liu et al.'s [11] study of Japanese patients due to undergo TKR, neither imaging or histology correlated with VAS or WOMAC scores. This is contrary to De Lange-Brokaar's [10] work that did show a correlation between MRI synovitis score and VAS assessment of pain. This is despite both studies finding a positive correlation between MRI findings and histological synovitis. Liu et al. [11] found only positive correlation between imaging and histology with the preoperative JKOM score. The reasons for this discrepancy are not clear. Most studies have found a correlation with more mainstream scoring systems such as VAS, WOMAC, KOOS, and the Intermittent and Constant Osteoarthritis Pain (ICOAP). Interestingly, Okuda et al.'s [21] earlier work had found no association between MRI synovitis score and pain. However, De Lange-Brokaar's earlier study had less study participants and included only patients due for arthroscopy rather than TKR.

Most studies identify that synovial tissue becomes more inflamed as osteoarthritis progresses in severity. With increasing OA severity, synovial membrane thickening, increased macrophage infiltration, and vascular proliferation are observed. However, Scanzello et al.'s study [24] has contradicted this finding. It is possible that differences in patient selection underlie this discrepancy. It is also possible that in Scanzello et al.'s study [24] the levels of inflammation were generally lower than in other studies. Ayral et al.'s [29] study shows evidence that the synovial inflammation profile changes depending on the level of OA present. Different patterns of synoviopathy with differences in tissue architecture and inflammatory infiltrates characterise distinct stages of OA. No studies commented on the presence of calcium pyrophosphate dihydrate (CPPD) crystal deposition either on haematoxylin and eosin staining or on polarised light microscopy. It is likely that at least some samples did show CPPD deposition disease given the often advanced state of joint degeneration and so CPPD cannot be ruled out.

With regard to cytokines and chemokine expression, there is less consensus within the literature. Many studies indicate increased levels of IL-1*α*, IL-1*β*, TNF*α*, and TGF*β* as OA progresses. However, there are other studies that have shown no difference in Interleukin levels with the exception of IL-15 [26] and IL-6 [27]. In another study, IL-1*β* levels decreased in end stage OA compared to progressive OA [25]. It is not clear why there is no consensus within the literature with regard to these findings. It may be due to differences in patient populations or possibly the variable use of medications. In samples from the same patient gene expression, patterns differ in tissues from inflamed and normal synovium, suggesting upregulation of key inflammatory pathways that could initiate or drive joint degeneration. Beyond the expression of cytokines, chemokines, and angiogenic compounds, it is argued that the presence of synovial inflammation has negative effects on cartilage integrity [19, 30].

To date, there is a paucity of studies showing how outcomes are related to synovial inflammation in the context of major joint replacement for OA. This is understandable since research into the inflammatory component of OA is a relatively new field. Only a few papers investigated pain or PROM scores and in all cases they were for preoperative assessments only. Perhaps it is assumed that since THR or TKR are definitive joint procedures then preoperative synovitis will play little or no part. Further research is required to confirm or refute this explanation.

## 5. Conclusion

In conclusion, synovitis is clearly present in OA, although it has not been determined if this is inherent to the disorder or a complication of secondary CPPD. The exact mechanisms are unknown and there is heterogeneity in the literature with respect to the levels of particular cytokines and inflammatory mediators within different stages of OA degeneration. Most studies report either “early” or “late” OA but it is not always clear what such a description means. Many studies also had low numbers of study participants. More research is needed with longitudinal studies applying proven imaging modalities, histological analysis, and longer follow-up. No studies have directly investigated the effect of synovitis on postoperative outcomes or compared the populations in which synovitis was more prevalent at the time of surgical intervention. It is possible that synovitis is related to other external factors and that this has an effect on clinical symptoms and outcomes. Research into this field is relatively new and will continue to define our understanding of the role of synovitis in the pathogenesis of OA.

## Figures and Tables

**Table 1:** Summary of the critical appraisal score (full papers only).

Study	CASP criterion number											
	1	2	3	4	5	6	7	8	9	10	11	Total
Benito et al., 2005 [22]	Y	Y	Y	Y	Y	N	Y	Y	Y	Y	Y	10
De Lange-Brokaar et al., 2014 [10]	Y	Y	Y	Y	Y	Y	Y	Y	Y	Y	Y	11
Lambert et al., 2014 [28]	Y	Y	Y	Y	Y	Y	Y	Y	Y	Y	Y	11
Liu et al., 2010 [11]	Y	Y	Y	N	N	N	Y	N	Y	Y	N	6
Oehler et al., 2002 [27]	Y	Y	N	Y	N	N	Y	Y	Y	Y	Y	8

Østergaard et al., 1998 [9]	Y	Y	Y	Y	Y	Y	Y	Y	Y	Y	Y	11
Scanzello et al., 2009 [24]	Y	Y	Y	Y	Y	Y	Y	Y	Y	Y	Y	11
Smith et al., 1997 [26]	Y	Y	N	Y	N	N	Y	Y	Y	Y	Y	8
Takase et al., 2012 [16]	Y	Y	N	N	N	N	Y	Y	Y	Y	Y	7
Tanavalee et al., 2011 [30]	Y	Y	Y	Y	N	N	Y	Y	Y	Y	N	8
Waldstein et al., 2014 [17]	Y	Y	Y	Y	Y	N	Y	Y	Y	Y	N	9
Walther et al., 2001 [15]	Y	Y	Y	Y	Y	Y	Y	Y	Y	Y	Y	11
Walther et al., 2002 [14]	Y	Y	Y	Y	Y	Y	Y	Y	Y	Y	Y	11

Y: yes; N: no.

(1) Did the study address a clearly focused issue?

(2) Was the cohort recruited in an acceptable way?

(3) Was the exposure accurately measured to minimize bias?

(4) Was the outcome accurately measured to minimize bias?

(5) Have the authors identified all important confounding factors?

(6) Have they taken account of the confounding factors in the design and/or analysis?

(7) Was the follow-up of subjects complete enough?

(8) Was the follow-up of subjects long enough?

(9) Do you believe the results?

(10) Can the results be applied to the local population?

(11) Do the results of this study fit with other available evidences?

**Table 2:** Study design and characteristics.

Study	OA/RA	Surgical intervention	Imaging modality	Synovial tissue scoring	Markers measured	Clinical scoring system	Result
Benito et al., 2005 [22]	OA	TKR or arthroscopy	XR	N/A	Inflammatory cell infiltrate, blood vessel formation, angiogenic factors, NF-kB activation, TNF*α*, IL-1*β*, COX-1, COX-2, and fibroblast like synoviocytes (FLS)	NA	Early OA tissue exhibited greater lining layer thickness and greater CD4+ T cell and CD68+ macrophage infiltration; also more numerous blood vessels and VEGF and ICAM-I expression and greater numbers of cells producing TNF*α* and IL-1*β*; FLS functionally similar in early and late OA

De Lange-Brokaar et al., 2013 (abstract) [18, 19]	OA	Arthroscopy	Contrast enhanced MRI	Guermazi et al. MRI scoring system	N/A	VAS pain	Knee synovitis severity on MRI correlated with macroscopic and microscopic histological synovitis features but not with VAS pain

De Lange-Brokaar et al., 2013 (abstract) [18, 19]	OA	Arthroscopy or TKR	Contrast enhanced MRI	Guermazi et al. MRI scoring system	N/A		Different patterns of knee synovitis were correlated to knee pain scoring systems

De Lange-Brokaar et al., 2014 [10]	OA	Arthroscopy or TKR	Gd-DOTA MRI 3T	Krenn et al. system, lining cell layer, synovial stroma, and inflammatory infiltrate graded 0–3	N/A	N/A	Synovitis severity on MRI correlated with macro- and microscopic features of synovitis in knee OA

Liang et al., 2009 (abstract) [23]	OA	TKR or arthroscopy or Anterior Cruciate Ligament (ACL) reconstruction		N/A	Synovial CD31, NF-xB, MMP-1, COX-2, IL-1*β*, and TGF-*β*	JKOM	Synovium MMP-1, COX-2, and IL-1*β* expression in end stage OA reduced in comparison to those of progressive OA; expression of TGF-*β* in the synovium of end stage OA was significantly increased in comparison to that of progressive OA; the MMP-1, IL-1*β*, and TGF-*β* expression in the synovium significantly correlated with the radiographic and clinical manifestations of knee OA

Lambert et al., 2014 [28]	OA	TKR	N/A	Surgeon graded macroscopic criteria by Ayral et al. normal, reactive, and inflamed	Cytokines, chemokines, enzymes, anabolism, catabolism, and angiogenesis compounds	N/A	Inflammation, cartilage metabolism, Wnt signalling, and angiogenesis pathways were upregulated in areas of inflamed synovium

Liu et al., 2010 [11]	OA	TKR	Gd-MRI 1.5T	Histological parameters 0–3	N/A	JKOM, VAS, and WOMAC	Neither synovitis nor Gd-MRI score correlates with pain VAS score or WOMAC; they only correlated with JKOM score

Oehler et al., 2002 [27]	OA	TKR or arthroscopy	N/A	Scored 0–4, immunohistochemistry	Inflammatory infiltrates	N/A	4 subtypes of synoviopathy characterised; hyperplastic in early OA; inflammatory in early and late OA; fibrotic and detritus rich in late OA

Østergaard et al., 1998 [9]	OA and RA	Knee arthroscopy or arthrotomy	Gd-DTPA MRI 1.5T	Histological parameters 0–3	N/A	N/A	Dynamic MRI can be used to determine synovial inflammation but could not differentiate between moderate and severe inflammation

Scanzello et al., 2009 [24]	OA	TKR	XR	N/A	IL-15, IL-6, IL-1*β*, TNF*α*, IL-2, IL-21, MMP-1, and MMP-3	N/A	Lymphocytic infiltrates found in all early OA specimens but only in some end stage specimens; IL-15 significantly higher in early OA patients

Smith et al., 1997 [26]	OA	TKR or arthroscopy	N/A	Scored 0–4, immunohistochemistry	IL-1*α*, IL-1*β*, TNF*α*, and IL-1ra	N/A	Synovial membrane inflammation more severe with advanced OA

Takase et al., 2012 [16]	OA and RA	TKR	GSUS, PDUS, and Gd-DTPA 1.5T MRI	OMERACT-RAMRIS (0–3), histopathology 0–3	Immunohistochemistry CD68, Ki-67, and CD31	N/A	GSUS, PDUS, and MRI correlated with pathological total synovitis score; only PDUS and MRI showed high correlation with immunohistochemical parameters (CD68 for inflammatory cell infiltrate, Ki-67 for synovial lining cell thickness, and CD31 for vascularity)

Tanavalee et al., 2011 [30]	OA	TKR	N/A	N/A	Serum CRP, ESR, and IL-6	American Knee Society score and knee surface temperature	Synovectomy at the time of TKR does not benefit the outcome nor shorten the duration of the inflammatory response

Walther et al., 2001 [15]	OA AND RA	TKR	Power Doppler sonography (PDS)	Vascularity graded 1–4	N/A	N/A	PDS reliable in qualitative grading of synovial tissue vascularity in knees

Walther et al., 2002 [14]	OA and RA	THR	PDS	Vascularity graded 1–4	N/A	N/A	PDS reliable in qualitative grading of synovial tissue vascularity in hips

Waldstein et al., 2014 [17]	OA	TKR	MRI 1.5 or 3T	WORMS scoring system; Krenn et al. synovium grading system, lining cell layer, synovial stroma, and inflammatory infiltrate graded 0–3	Synovial WBC count, Mankin cartilage grading system; aggregate and dynamic modulus cartilage biomechanics	N/A	Cartilage mechanically inferior in knees with WBC >150 WBCs/mL compared to knees with <150 WBCs/mL; MRI and histopathologic synovitis grades did not correlate with each other or aggregate or dynamic cartilage modulus

**Table 3:** Genes differentially expressed between normal/reactive and inflamed areas of synovium.

Compound	Upregulated	Downregulated
Inflammatory cytokines	IL8, IL6, TNFRSF21, IFI30, TNFAIP6, and IRF8	

Inflammatory chemokines	CXCL5, CXCL6, CXCL16, CXCL2, and CXCL1	

Inflammatory enzymes	ALOX5AP, PLD1, ALOX5, PTGES, PLCB1, SOD2, TBXAS1, PI3, and PLA2G4A	

Other inflammatory compounds	TREM1, S100A9, OSM, and PPARG	

Anabolism	HAS1, BMP6, and COLL22A1	COL1A2, VIM, MATN2, HABP4, HAPLN1, HAS3, COL16A1, CILP, COL6A3, GPC4, HAPLN1, and ACAN

Catabolism	MMP9, MMP3, CTSH, ADAMDEC1, and CTSS	

Angiogenesis	STC1, PF4V1, EDNRB, AQP9, HBEGF, BDKRB1, RCAN1, ECGF1, DNER, BDKRB2, and PECAM1	PDGFC and RNH1

IL: Interleukin, TNFRS: tumour necrosis factor receptor, and IFI30: gene for encoding Gamma-interferon-inducible lysosomal thiol reductase. TNFAIP6: tumor necrosis factor alpha-induced protein 6, IRF: interferon regulatory factor, CXCL: chemokine ligand, and ALOX5AP: 5-lipoxygenase activating protein encoded by the ALOX5AP gene. PLD: phospholipase D1 enzyme that is encoded by the PLD1 gene, PTGES: prostaglandin E synthase is an enzyme encoded by the PTGES gene, PLCB1: 1-phosphatidylinositol-4,5-bisphosphate phosphodiesterase beta-1 is an enzyme encoded by the PLCB1 gene, and SOD2: superoxide dismutase 2 enzyme encoded by the SOD2 gene. TBXAS1: thromboxane A synthase 1 enzyme encoded by the TBXAS1 gene, PI3: elafin, PLA2G4A: cytosolic phospholipase A2 enzyme encoded by the PLA2G4A gene, TREM1: triggering receptor expressed on myeloid cells 1 protein encoded by the TREM1 gene, S100A9: migration inhibitory factor-related protein 14, OSM: oncostatin M, PPARG: peroxisome proliferator-activated receptor gamma, HAS1: hyaluronan synthases 1, BMP: bone morphogenetic protein, COL: collagen, VIM: vimentin, MATN: cartilage matrix protein, HABP: hyaluronan-binding protein, HAPLN: proteoglycan link protein, HAS: hyaluronan synthase, CILP: intermediate layer protein, GPC: cerebroglycan, ACAN: aggrecan, MMP: metalloproteinase, CTSH: cathepsin H, ADAMDEC1: ADAM-like, decysin 1, CTSS: cathepsin S, STC1: stanniocalcin-1, EDNRB: endothelin receptor type B, AQP9: aquaporin-9, HBEGF: heparin-binding EGF-like growth factor, BDKRB1: bradykinin receptor B1, RCAN1: Down syndrome critical region gene 1, ECGF1: platelet-derived endothelial cell growth factor, DNER: Delta and Notch-like epidermal growth factor-related receptor, BDKRB2: bradykinin receptor B2, PECAM1: platelet endothelial cell adhesion molecule, PDGFC: platelet-derived growth factor C, and RNH1: ribonuclease inhibitor.
